# Parental Knowledge on Prevention of SUID: Dziyana Nazaruk

**DOI:** 10.1093/eurpub/ckac131.427

**Published:** 2022-10-25

**Authors:** D Nazaruk, A Palacios, N Marshall, J Choongo

**Affiliations:** Health Policy and Community Health, Georgia Southern University, Savannah, USA

## Abstract

**Background:**

According to the 2018 America’s Health Ranking Annual Report, the U.S. infant mortality rate (IMR) was 5.9 deaths per 1,000 live infant births, which placed the U.S. at No. 33 out of 36 countries with the world’s largest economies in 2018. The IMR in Georgia has been on the rise since 2014. Even though there was a sharp decline in sudden unexpected/unexplained causes of death (SUID) beginning in 1990, the decrease has considerably slowed down since 1999. In 2017, the top ten leading causes of infant death were responsible for 67.8% of all infant deaths in the United States. Sudden Infant Death Syndrome (SIDS) and death from unintentional injuries were in third and fourth place. Therefore, the purpose of this research is to utilize qualitative research guided by the Socio-Ecological model to understand better how prepared first-time mothers are to take care of their infant upon hospital release.

**Methods:**

An intensity sampling was utilized to recruit participants. The inclusion criteria for the participants included first-time mothers with children under one year of age, women, Georgia residents over 18 years of age, and English speaking. A semi-structured guide was based on research queries and the ideological concepts of the Socio-Ecological Model. For the qualitative data examination, the thematic analysis was performed. All the interviews were transcribed verbatim and coded by using the program NVivo 11.

**Results:**

A total of 25 women participated in the study. We will finish the data analysis by the end of May, 2022. The results of this qualitative study will help to fully understand the knowledge, perceptions, skills, and confidence of mothers about infant care.

**Conclusions:**

The study’s results will help develop an information guide on primary infant care. Besides, health professionals and community organizations can utilize the study results to determine the information and support needed for new mothers.

**Key messages:**

• The results of this qualitative study will help to fully understand the knowledge, perceptions, skills, and confidence of mothers about infant care.

• This study’s results will help develop an information guide (electronic or/and printed) on primary infant care based on first-time mothers’ needs.

